# BMP9 Is a Proliferative and Survival Factor for Human Hepatocellular Carcinoma Cells

**DOI:** 10.1371/journal.pone.0069535

**Published:** 2013-07-23

**Authors:** Blanca Herrera, María García-Álvaro, Silvia Cruz, Peter Walsh, Margarita Fernández, Cesáreo Roncero, Isabel Fabregat, Aránzazu Sánchez, Gareth J. Inman

**Affiliations:** 1 Dep. Bioquímica y Biología Molecular II, Facultad de Farmacia, Universidad Complutense de Madrid, Instituto de Investigación Sanitaria del Hospital Clínico San Carlos (IdISSC), Madrid, Spain; 2 Division of Cancer Research, Medical Research Institute, Ninewells Hospital and Medical School, University of Dundee, Dundee, United Kingdom; 3 Bellvitge Biomedical Research Institute (IDIBELL) and University of Barcelona (UB), ĹHospitalet de Llobregat, Barcelona, Spain; University of Navarra School of Medicine and Center for Applied Medical Research (CIMA), Spain

## Abstract

TGF-β family members play a relevant role in tumorigenic processes, including hepatocellular carcinoma (HCC), but a specific implication of the Bone Morphogenetic Protein (BMP) subfamily is still unknown. Although originally isolated from fetal liver, little is known about BMP9, a BMP family member, and its role in liver physiology and pathology. Our results show that BMP9 promotes growth in HCC cells, but not in immortalized human hepatocytes. In the liver cancer cell line HepG2, BMP9 triggers Smad1,5,8 phosphorylation and inhibitor of DNA binding 1 (Id1) expression up- regulation. Importantly, by using chemical inhibitors, ligand trap and gene silencing approaches we demonstrate that HepG2 cells autocrinely produce BMP9 that supports their proliferation and anchorage independent growth. Additionally, our data reveal that in HepG2 cells BMP9 triggers cell cycle progression, and strikingly, completely abolishes the increase in the percentage of apoptotic cells induced by long-term incubation in low serum. Collectively, our data unveil a dual role for BMP9, both promoting a proliferative response and exerting a remarkable anti-apoptotic function in HepG2 cells, which result in a robust BMP9 effect on liver cancer cell growth. Finally, we show that BMP9 expression is increased in 40% of human HCC tissues compared with normal human liver as revealed by immunohistochemistry analysis, suggesting that BMP9 signaling may be relevant during hepatocarcinogenesis *in vivo*. Our findings provide new clues for a better understanding of BMPs contribution, and in particular BMP9, in HCC pathogenesis that may result in the development of effective and targeted therapeutic interventions.

## Introduction

Aberrant Transforming Growth Factor–beta (TGF-β) signaling has been associated with many human diseases, including cancer [1]. During the tumorigenic process, TGF-β acts as a double edge-sword: on one hand, in normal and pre-malignant cells, TGF-β is a cytostatic, pro-apoptotic factor. On the other hand, in cancer cells, TGF-β promotes tumor growth, immune evasion and a migratory/invasive phenotype. What roles other TGF-β superfamily members, such as the Bone Morphogenetic Protein (BMP) subfamily play in cancer, has only been started to be addressed in the past few years.

BMPs were discovered about 50 years ago for their capacity to regulate bone and cartilage formation [2], [3], but our current knowledge indicates BMPs have a much broader role than originally thought [4]. In fact, their relevant function during development is beyond any reasonable doubt [5], and in recent years, it has become clear that BMPs also play a significant role in adult tissue homeostasis by controlling many different cellular processes such as proliferation, differentiation, migration, survival and apoptosis [4], [6]. BMPs exert their effects by binding to a heterotetrameric transmembrane receptor complex formed by a type I (Activin like kinase, ALK) and a type II serine/threonine kinase receptor that once activated recruits and phosphorylates the specific effector proteins, so-called R-Smads, Smad1, 5 and 8. Phosphorylated Smad1,5,8 bind to co-Smad Smad4 and accumulate in the nucleus, where together with specific binding partners modulate gene transcription [7]. It has been described that BMPs could also trigger non-canonical or non-Smad signaling pathways that in certain contexts are key for the biological effects of BMPs [8].

Dysregulation of BMP signaling can have pathological consequences in many different diseases [9]. Among them, BMP contribution in cancer is a matter of intense investigation as both pro- and anti-tumorigenic activities for different members of the family have been reported [10], [11], [12]. BMP ligands are overexpressed in several tumor types, including prostate, melanoma, non-small cell lung carcinoma, ovarian and gastric cancer [12]. Besides, aberrant expression of BMP receptors has been associated with the tumorigenic process [11]. Pro-tumorigenic activity of the BMPs not only includes promotion of proliferation, migration/invasion, epithelial to mesenchymal transition (EMT) and survival, but recently their role in tumor dormancy and recurrence has been described [13].

Hepatocelullar carcinoma (HCC) is a devastating cancer type, ranking sixth in incidence and the third leading cause of cancer death worldwide, yet molecular mechanisms driving hepatocarcinogenesis remain largely unknown [14]. In fact, the role of BMPs in HCC has only began to be addressed [15]. Aplying the systems biology approach, in hepatitis B virus X antigen transgenic mouse, BMP7 and BMP4 have been found to be up-regulated in cirrhosis and liver cancer [16]. Furthermore, BMP4 and BMP6 are overexpressed in both human HCC cell lines and tissues and importantly, BMP4 expression strongly correlates with high tumor grade [17], [18] having recently being proposed as marker for the prediction of HCC recurrence and prognosis [19]. BMP4 produced by HCC cells has an autocrine effect promoting invasion and anchorage independent growth, together with a paracrine effect increasing tube formation in endothelial cells, thus promoting tumor vasculogenesis. BMP antagonists, Noggin and Chordin, which are capable of sequestering BMP ligands in the extracellular space, impaired HCC cell migration and invasion confirming the BMP signaling involvement in these cellular processes [20]. BMP9 was first isolated in fetal mouse liver [21], and it is also expressed in adult healthy liver [22]. Although it is known to be overexpressed in Hep3B and PLC/PRF/5 HCC cells [20], its precise contribution to the hepatocarcinogenesis process has just began to be explored. In a recent work, Li and coworkers have found that BMP9 induces EMT and increased migration in HCC cells [23]. Here, we have investigated BMP9 role in HCC cell growth. Our results provide solid evidence for a role of BMP9 in the promotion of HCC cell growth, effect that was not observed in non-transformed hepatocytes. In HepG2 cells, we demonstrated that BMP9 promotes survival and both anchorage dependent and independent cell growth. Importantly, using different approaches we also show that HepG2 cells presented an autocrine BMP9 production that might occur also *in vivo*. Altogether, our findings provide new clues for a better understanding of BMP contribution in HCC pathogenesis.

## Materials and Methods

### 1. Materials

The following reagents were used: BMP9 and ALK1 extracellular domain (ALK1ecd) were from R&D Systems (Minneapolis, MN). Dorsomorphin was from Calbiochem (La Joya, CA) and LDN193189 from Miltenyi Biotec (Pozuelo de Alarcón, Madrid). AccuMax tissue microarrays of liver cancer tissues (A204II) and ovary cancer tissues (A213II) were purchased from Stretton Scientific Ltd. Bronchial Epithelial Cell Growth Medium (BEBM) and BEGM bullet kit were purchased from Lonza Iberica (Barcelona, Spain). Dulbecco’s modified Eagle’s medium (DMEM), Minimum Essential Medium (MEM), fetal bovine serum (FBS) and trypsin-EDTA were from Gibco-Invitrogen (Barcelona, Spain). Penicillin, streptomycin, HEPES, bovine serum albumin (fraction V, fatty-acid free), propidium iodide and all buffer reagents were from Sigma-Aldrich. [^3^H]-thymidine (25.0 Ci/mmol), Horseradish peroxidase-conjugated secondary antibodies and ECL reagent, were from GE Healthcare Europe (Barcelona, Spain). Antibodies against the following proteins were used: Phospho-Smad1 (Ser463/465)/Smad5 (Ser463/465)/Smad8 (Ser426/428) polyclonal antibody (#9511) and Smad1 polyclonal antibody (#9743) from Cell Signaling Technology (Beverly, MA). Smad2/3 monoclonal antibody (#610842) was from BD Biosciences (Madrid, Spain) and Inhibitor of DNA binding1 (Id1) (C-20) polyclonal antibody (sc-488) from Santa Cruz Biotechnology, Inc. (Paso Robles, CA). All of them were used 1∶1000. Polyclonal anti human BMP9 antibody (AP2064a, Abgent, San Diego, CA) was used in immunohistochemical analysis.

### 2. Cell Culture

HepG2, Hep3B and Huh7 human HCC epithelial cells were obtained from the European Collection of Cell Cultures (ECACC), and non-tumoral human hepatocyte cell line THLE3 from the American Type Culture Collection (ATCC). For cell culture, the following media were used: DMEM for HepG2 and Huh7, MEM for Hep3B and BEBM supplemented with BEGM Bullet kit for THLE3. THLE3 cells were cultured in coated plates (0.01 mg/ml fibronectin, 0.03 mg/ml collagen I and 0.01 mg/ml BSA). HepG2 cells stably expressing a reporter plasmid consisting of BMP-responsive elements from the Id1 promoter fused to a luciferase reporter gene (HepG2BRA) were kindly provided by Dr. Rifkin, from New York University Langone School of Medicine [24]. HepG2BRA cells were cultured in DMEM supplemented with 700 µg/ml G418. Cell lines were grown in media supplemented with 10% FBS, 100 U of penicillin and streptomycin per ml and maintained in a humidified incubator at 37°C and a 5% CO_2_ atmosphere.

### 3. Proliferation Studies

10,000 or 20,000 cells/well in 12 well plates were plated and serum starved prior to treatment with different factors. At various time points, cells were harvested by trypsinization and cell number was determined using a Casy cell counter (Roche).

### 4. DNA Synthesis Analysis

Cells were plated at a density of 17,500 cells/sq cm in DMEM with 10% FBS. The following day, cells were serum starved and incubated for 48 hours with or without BMP9. Incorporation of [^3^H]-thymidine during the last 40 hours of culture was measured in trichloroacetic acid-precipitable material as previously described [25].

### 5. Analysis of Cell DNA Content by Flow Cytometry

Cells were harvested by trypsinization, fixed in 70% ethanol (−20°C) for 1 min, and treated with RNaseA (10 µg/ml) for 30 min at 37°C. After propidium iodide staining (0.05 mg/ml, 15 min at room temperature in the dark), the cellular DNA content was analyzed in a FACScan flow cytometer (Becton-Dickinson, San Jose, CA). For computer analysis, only signals from single cells were considered (10,000 cells/assay).

### 6. Analysis of Apoptosis by Phosphatidylserine Exposure

Cells were harvested by trypsinization and washed once with PBS. 500,000 cells were resuspended with 195 µl of binding buffer (10 mM HEPES, pH 7.4, 2.5 mM CaCl_2_, 140 mM NaCl) supplemented with 5 µl annexin V-FITC (BD Pharmingen) and incubated for 10 min at room temperature. Samples were centrifuged and resuspended with 300 µl of binding buffer containing 1 µg/ml propidium iodide. Fluorescence intensity was analyzed using a FACSCalibur flow cytometer. 10,000 cells were recorded in each analysis.

### 7. Measurement of Apoptotic Index

Measurement of apoptotic index was performed as previously described [26]. After staining with propidium iodide, cells undergoing apoptosis were scored under inverted fluorescence microscope (Eclipse TE300, Nikon) at high magnification (x60) following standard morphological criteria. Apoptotic indices were calculated after counting a minimum of 1000 cells per treatment in a blinded manner.

### 8. Western Blotting

Whole cell lysates and western blotting was performed as described previously [27].

### 9. RNA Isolation and qRT-PCR

RNA was isolated using TRIZOL reagent (Invitrogen). cDNA was prepared using the DyNAmo Sybr Green 2-step qRT-PCR kit (Finnzymes). Quantitative RT-PCR was performed as described [27]. Id1, BMP9 and 18S primers were obtained from Qiagen. Amplified products were analyzed by a Chromo4 continuous fluorescence detector (Biorad) and Opticon Monitor3 software.

### 10. Transcriptional Reporter Assay

20,000 HepG2BRA cells were plated in 24 well plates containing DMEM plus 10% FBS and allowed to attach for 18 hours. Cells were incubated with DMEM 0.1% FBS for 8 hours and BMP9 added. After 15 hours of treatment, cells were washed with PBS and lysed using 100 µl of reporter lysis buffer (Promega, Madison, USA). To measure luciferase activity, 40 µl of lysate were added to 40 µl Luciferase Assay Reagent (Promega) and luminescence was quantitated using a Fluostar Omega luminometer (RMG Labtech). Luciferase units were normalized per cell number.

### 11. Soft Agar Assays

Soft agar assays were performed as previously described [28]. Briefly, 20,000 cells/well in 6 well plates were plated in DMEM supplemented with 5% FBS and 0.45% agarose on the top of solidified agarose (0.9% in DMEM supplemented with 5% FBS). 300 µl DMEM with or without the treatment were added to each well twice weekly. Colonies were counted 3 weeks after seeding. Colonies of more than 50 µm in diameter were scored.

### 12. Retroviral Infection

Oligonucleotides targeting human BMP9 or non-silencing oligos (Table S1 in File S1) were annealed and cloned into *Xho-1*/*Eco-RI* digested MSCV/LTRmiR30-PIGΔRI (LMP) (a kind gift of Ross Dickins and Scott Lowe). All constructs were sequenced prior to use and are referred to as non-silencing (LMP-NS), LMP-shBMP9#1 and LMP-shBMP9#2. Retrovirus was generated as described [27]. Stable cell pools were generated after outgrowth in media containing 0.5 µg/ml puromycin.

### 13. Immunohistochemistry

Immunohistochemistry with BMP9 antibody was performed as previously described [27].

### 14. Statistical Analysis

Statistical analysis was performed by Student’s *t*-test analysis.

## Results

### 1. BMP9 Promotes Cell Growth in HCC Cell Lines but not in Non-transformed Hepatocytes

**Figure 1:**
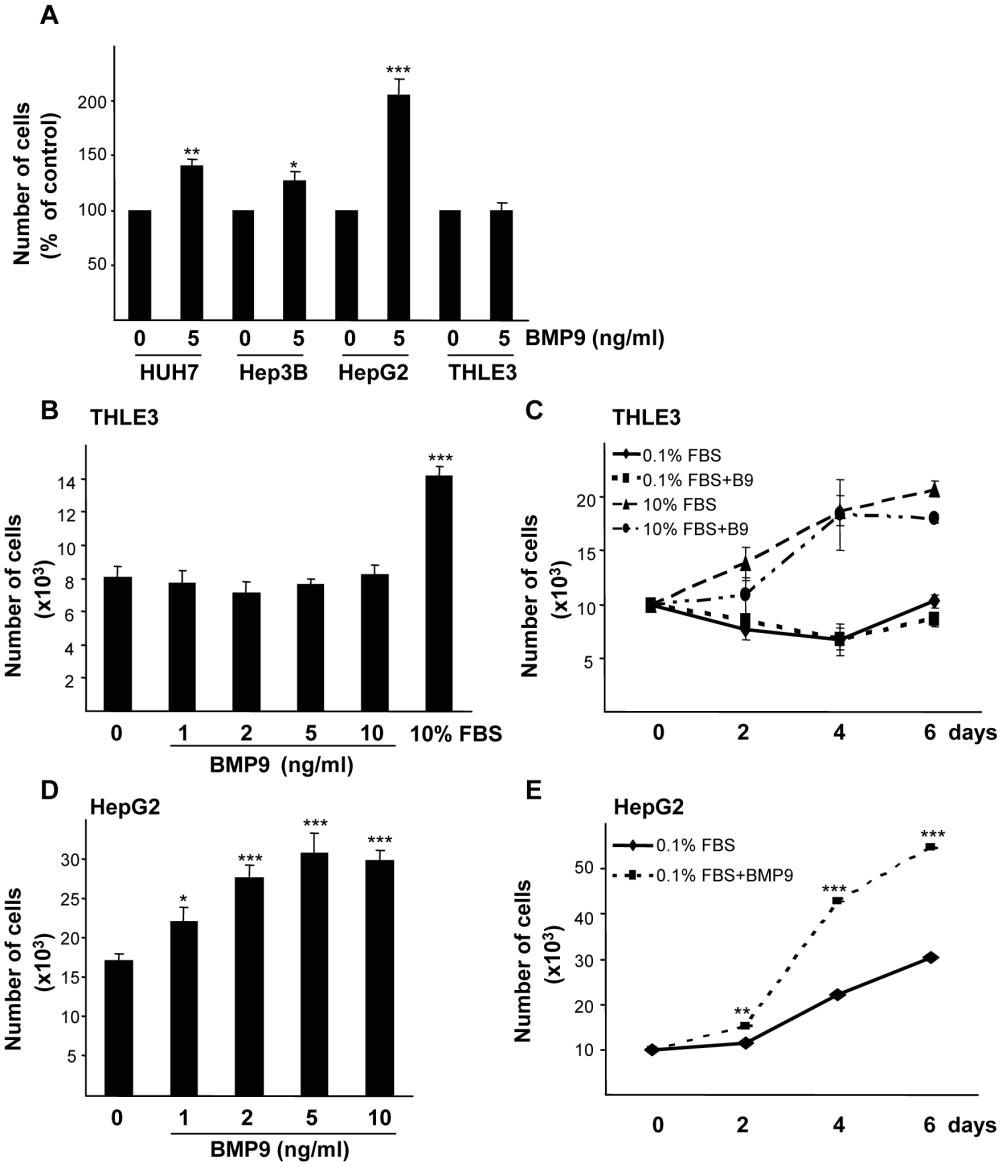
BMP9 increases cell number of HCC cell lines but not of immortalized human hepatocytes. **A.** Huh7, Hep3B and HepG2 liver cancer cells and immortalized human hepatocytes (THLE3) were incubated in the absence or in the presence of 5 ng/ml BMP9 in 0.1% FBS media and counted at day 4. Data from 3 independent experiments performed in triplicate (mean ± S.E.M.) are shown. **B.** THLE3 cells were incubated with different concentrations of BMP9 in 0.1% FBS media and counted after 4 days of treatment. Data from 3 independent experiments performed in triplicate (mean ± S.E.M.) are shown. **C.** Proliferation curve of THLE3 cells incubated for different periods of time −/+ BMP9 (5 ng/ml) in 0.1% FBS or in 10% FBS media. Data from one representative experiment (n = 3) out of 3 (mean ± S.D.) are shown. **D.** HepG2 cells were incubated with different concentrations of BMP9 in 0.1% FBS media and counted after 4 days of treatment. Data from 3 independent experiments performed in triplicate (mean ± S.E.M.). **E.** HepG2 cells were incubated for different periods of time −/+ BMP9 (5 ng/ml) in 0.1% FBS media. Data from 6 independent experiments performed in triplicate (mean ± S.E.M.). Statistical analysis was carried out using the paired *t-*test and data were compared to untreated samples, * = *P*<0.05, ** = *P*<0. 01, *** = *P*<0.001.

Previous reports in the literature have indicated that BMP9 is expressed mainly in fetal [21] and adult [22], [29] liver where it was shown to bind membrane receptors in non-parenchymal cells, particularly liver endothelial cells and Kupffer cells [29]. The HCC cell line HepG2 was also shown to respond to BMP9 with an increased proliferation [30]. In order to extend these observations, we first analyzed the response to BMP9 in terms of cell growth of different liver cancer cell lines. The effect of BMP9 was checked in low serum conditions (0.1% FBS) to avoid the bioactive concentrations of BMPs (BMP4, BMP6 and importantly, BMP9) in FBS [31]. Therefore, HepG2, Hep3B and Huh-7 human HCC cells were incubated for 15 hours in 0.1% FBS media and then treated with 5 ng/ml of BMP9 for 4 days. BMP9 triggered a moderate but significant growth stimulatory effect in Huh7 and Hep3B cells, while HepG2 cell number was doubled upon BMP9 treatment (figure 1A). Importantly, when non transformed hepatocytes, such as primary adult mouse hepatocytes, immortalized mouse neonatal hepatocytes or a human immortalized hepatocyte cell line (THLE3) were treated with BMP9 in the same conditions, the proliferative effect of BMP9 was not observed, and even a decrease in cell number was found in primary hepatocytes (figure 1A and figure S1 in File S1). To further confirm these data, THLE3 cells were incubated with different BMP9 concentrations and yet again, cell numbers were similar in all conditions, whilst cells did respond to proliferative stimulus like 10% FBS (figure 1B). We next assayed BMP9 treatment for different periods of time, in the presence of 10% FBS or in low serum conditions (0.1% FBS), and THLE3 cells did not respond to BMP9 in terms of growth in any of the conditions tested (figure 1C). As HepG2 cells presented the best response to BMP9-induced cell growth, we decided to use them for subsequent experiments. In order to further optimize, if possible, our experimental settings, we next performed a dose/response analysis of the growth effect in HepG2 cells using two different approaches, cell counting and the CellTiter-Glo luminescent cell viability assay. BMP9 increased cell number in a dose-dependent manner, having a maximal response at concentration of 5 ng/ml that was used hereafter (figure 1D and figure S2A in File S1). Additionally, we performed a time-course analysis of the BMP9 growth effect at different time points. After 2 days of treatment, a significant difference in cell number between non-treated and treated cells was found, which was greater after longer treatment (4 to 8 days), with a 2- to 4-fold increase in the number of cells (figure 1E and figure S2B in File S1). To know the relevance of the BMP9 growth effect in HepG2 cells, we compared it with other well-known liver mitogens such as EGF, insulin and IGF1. Our data revealed that BMP9-promoted HepG2 cell growth was comparable to that observed with insulin, IGF1 and EGF at their respective optimal concentrations (figure S3 in File S1). All together these data suggest that BMP9 promotes cell growth of liver cancer cell lines, an effect that is not observed in non-transformed hepatocytes.

### 2. BMP9 Activates the Canonical Pathway in HepG2 Cells

**Figure 2:**
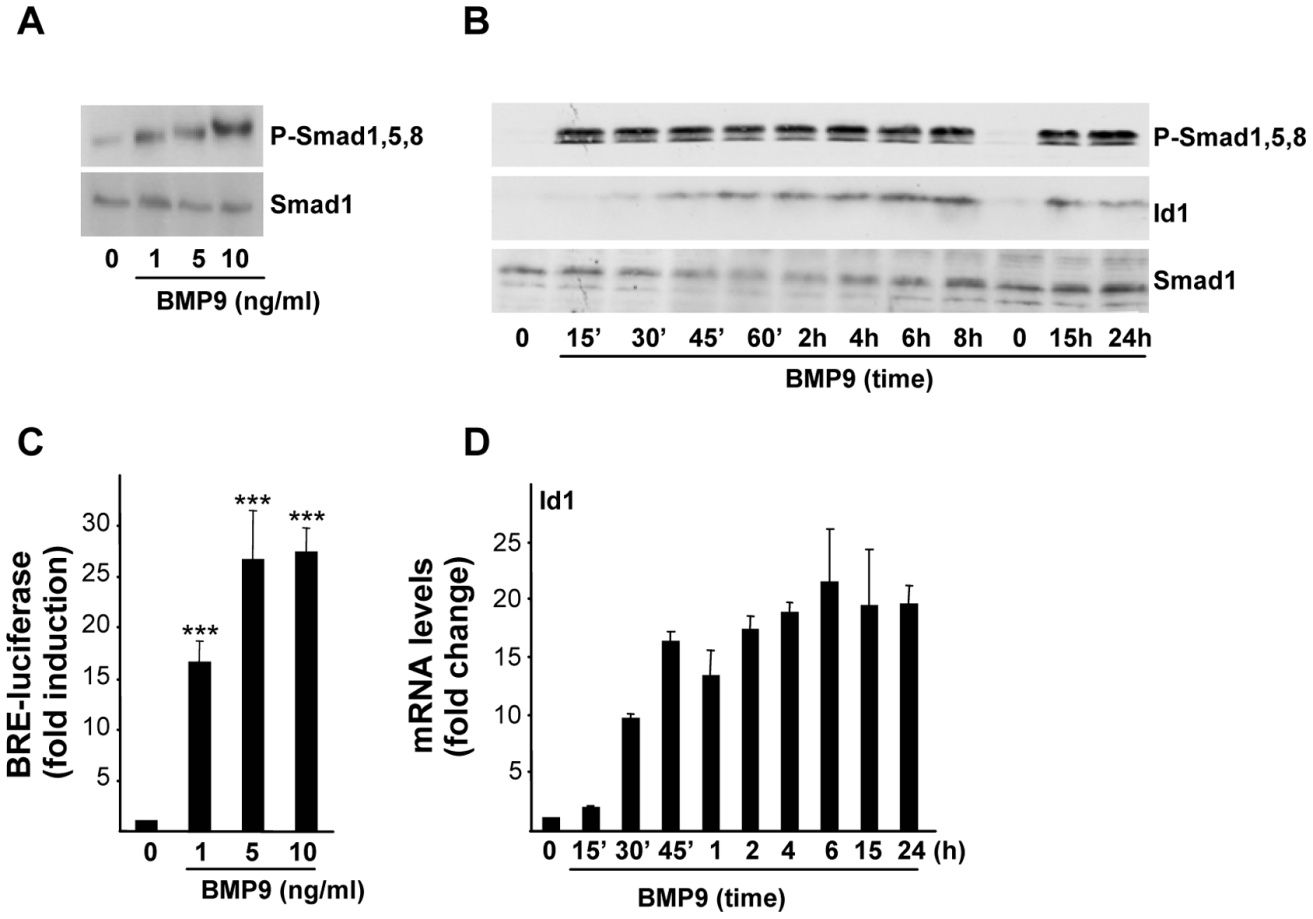
BMP9 activates the Smad1,5,8 pathway in HepG2 cells. **A.** HepG2 cells were incubated for 1 hour with different concentrations of BMP9 in 0.1% FBS media. Western blots were performed with antibodies that recognized activated (phosphorylated) Smad1, 5 and 8 (P-Smad1,5,8) and Smad1 as loading control. A representative experiment of 2 is shown. **B.** HepG2 cells were incubated for different periods of time −/+ BMP9 (5 ng/ml) in 0.1% FBS media. Western blots were performed with antibodies that recognize Id1, P-Smad1,5,8 and total Smad1 (loading control). A representative experiment of 3 is shown. **C.** HepG2 stably expressing BRE-luciferase (HepG2-BRA) cells were plated and incubated with 0.1% FBS for 15 hours, and then treated with different concentrations of BMP9 for additional 15 hours. Luciferase activity was normalized to cell number. Data are shown as fold induction (relative to untreated cells) and are from one representative experiment (n = 4) out of 3 performed (mean ± S.D.). Statistical analysis was carried out using the paired *t*-test and data were compared to untreated samples, *** = *P*<0.001. **D.** HepG2 cells were incubated −/+ BMP9 (5 ng/ml) for different periods of time in 0.1% FBS media and Id1 levels were analyzed by qRT-PCR and normalized to 18S. Fold changes relative to untreated samples were determined (mean ± S.E.M, n = 3).

We next wanted to analyze the signaling pathway triggered by BMP9 in HepG2 cells. BMP9 induced Smad1,5,8 phosphorylation in a dose-dependent manner (figure 2A). Phosphorylation of Smad1,5,8 occurred early, being already observed after 10–15 minutes of BMP9 treatment, and it was sustained for over at least 24 hours (figure 2B). To test the transcriptional response to BMP9, we used HepG2 cells stably expressing a reporter construct consisting of a BMP-responsive element (BRE) from the Id1 promoter fused to a luciferase reporter gene, named as HepG2BRA cells [24]. These cells have been previously shown to respond to several BMPs including BMP2, BMP4, BMP6 and BMP7 by increasing the luciferase activity [24]. Here, HepG2BRA cells were challenged with different concentrations of BMP9 and the induction of luciferase expression was analyzed. Data presented in figure 2C show that BMP9 treatment increased BRE-luciferase reporter activity in a dose-dependent manner with a maximal stimulation at a concentration of 5 ng/ml. Consistent with these results, we found that BMP9 enhanced Id1 expression, both at mRNA and protein levels (figure 2D and 2B, respectively). Jointly, these data indicate that BMP9 triggers the canonical Smad1,5,8 pathway in HepG2 cells.

### 3. Autocrine BMP9 Production Supports Anchorage Dependent and Independent HepG2 Cell Growth

**Figure 3:**
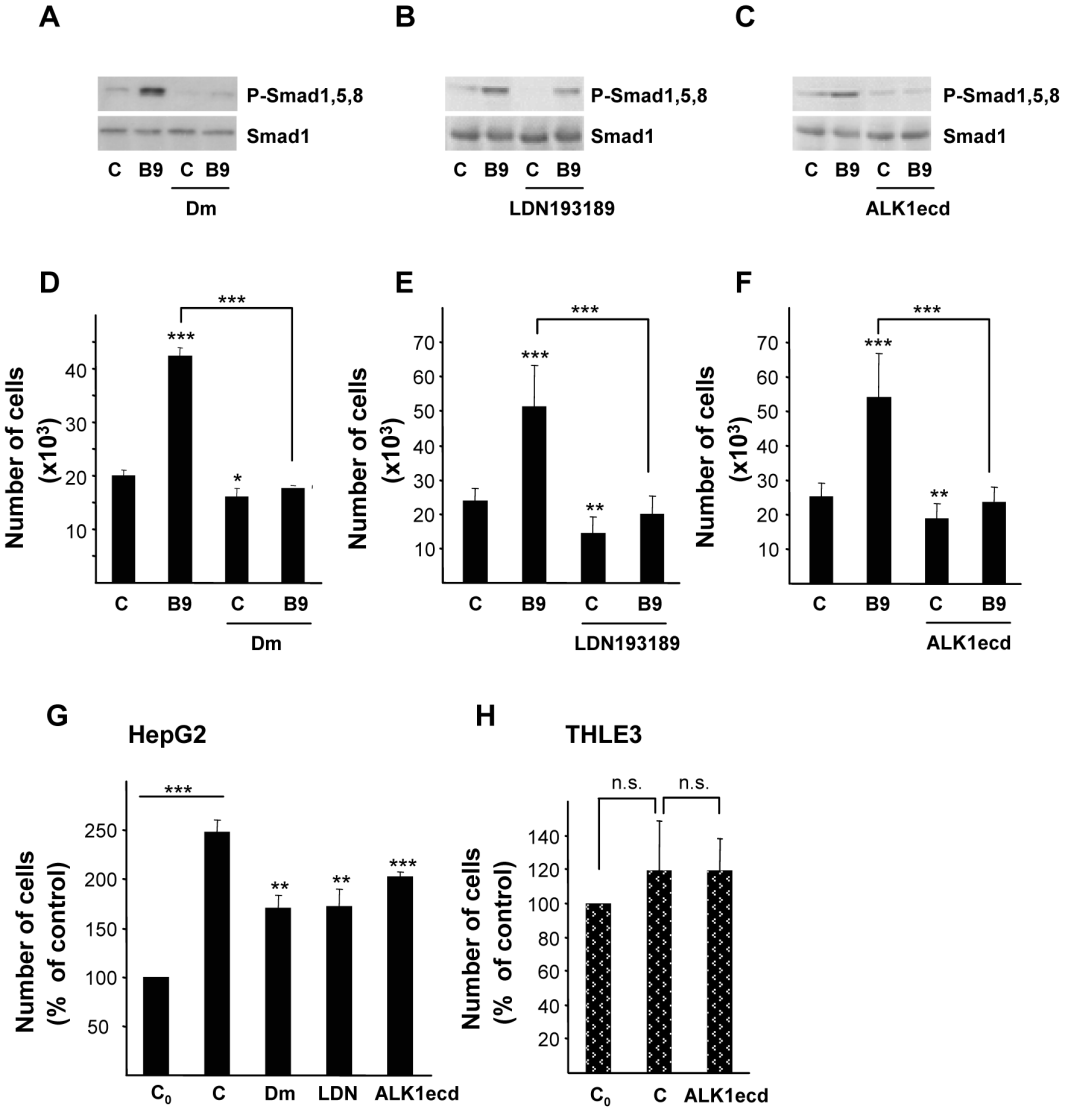
Effect of BMP receptor inhibitors and ALK1ecd treatment on HepG2 cell growth. **A, B and C.** HepG2 cells were incubated for 1 hour with **A.** dorsomorphin (1 µM, Dm), **B.** LDN193189 (100 nM) or **C.** ALK1ecd (16 fold molar excess, F.M.E.) and −/+ BMP9 (5 ng/ml) in 0.1% FBS media. Western blots were performed with antibodies that recognize P-Smad1,5,8 and Smad1 as loading control. A representative experiment of 2 is shown in each case. **D.** HepG2 cells were incubated as in **A** and counted at day 4. Data from 2 independent experiments performed in triplicate (mean ± S.E.M.). **E.** HepG2 cells were incubated as in **B** and counted at day 4. Data from 3 independent experiments performed in triplicate (mean ± S.E.M.). **F.** HepG2 cells were incubated as in **C** and counted at day 4. Data from 3 independent experiments performed in triplicate (mean ± S.E.M.). **G. **HepG2 cells were incubated without (C) or with dorsomorphin (Dm, 1 µM), LDN193189 (100 nM) or ALK1ecd (16 F.M.E) in 0.1% FBS media and counted at day 4. Data from at least 3 independent experiments performed in triplicate, displayed as percentage of C_0_ samples (untreated cells, day = 0) (mean ± S.E.M). **H.** THLE3 cells were incubated with ALK1ecd (16 F.M.E) in 0.1% FBS media and counted at day 4. Data from 2 independent experiments performed in triplicate, displayed as percentage of C_0_ (untreated cells, day = 0). Statistical analysis was carried out using paired *t*-test and data were compared to untreated samples, * = *P*<0.05, ** = *P*<0. 01, *** = *P*<0.001 or as indicated. n.s. = not significant.

**Figure 4:**
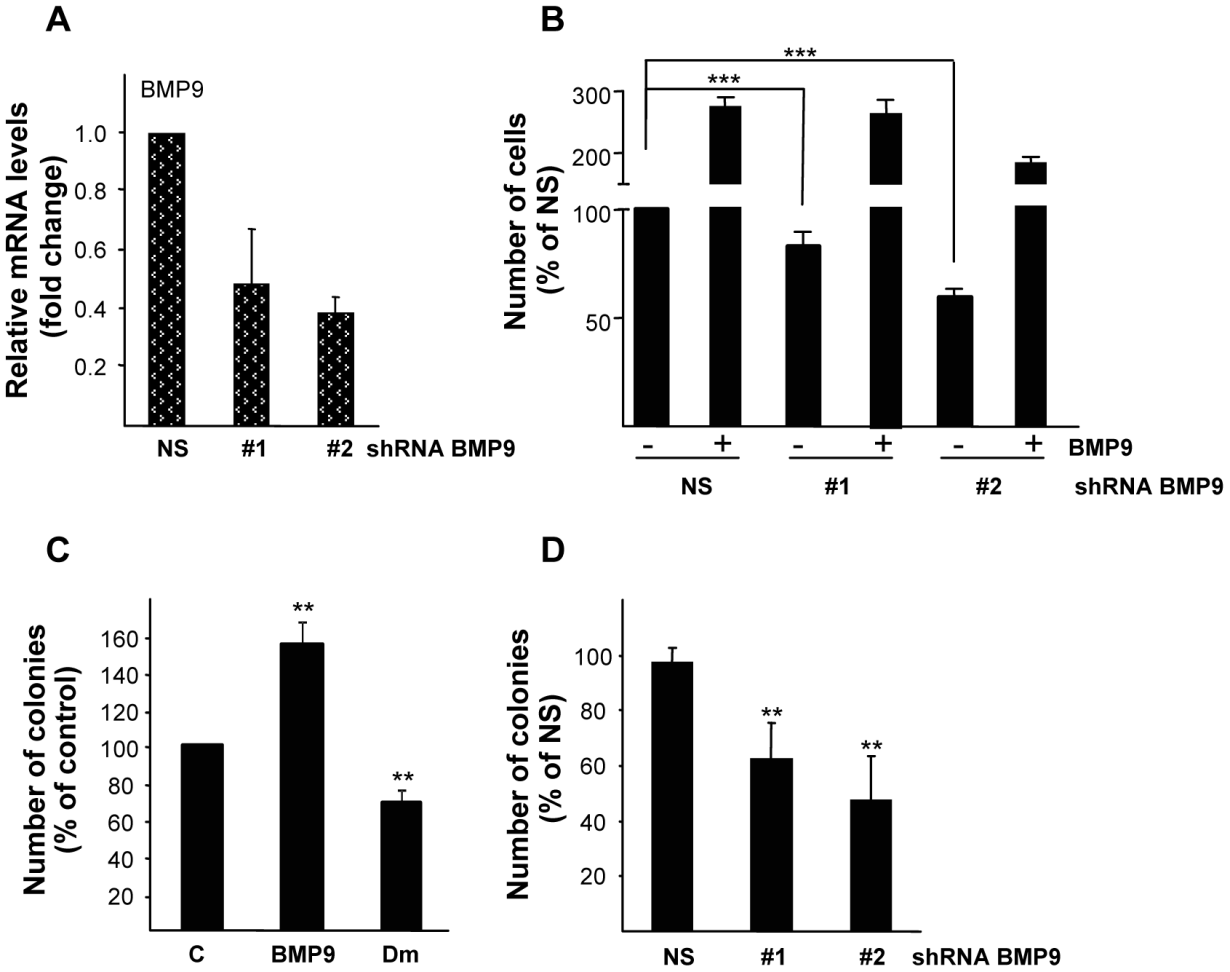
BMP9 production supports HepG2 anchorage dependent and independent cell growth. **A.** Independent stable cell lines expressing non-silencing (N.S.) and two different shRNAs targeted against BMP9 were generated by retroviral infection of HepG2 cells. BMP9 mRNA levels were determined by quantitative RT-PCR and normalized to 18S. Data expressed relative to N.S. cells (assigned an arbitrary value of 1) from 3 different experiments (mean ± S.E.M). **B.** Non-silencing (NS), shBMP9#1 and #2 stable HepG2 cell lines were incubated in 0.5% FBS and −/+ BMP9 (5 ng/ml) and counted at day 6. Data from 6 independent experiments performed in triplicate, displayed as percentage of N.S. untreated cells (mean ± S.E.M). **C.** HepG2 cells were plated in soft agar and treated with BMP9 (5 ng/ml) or with dorsomorphin (Dm, 1 µM) for 3 weeks (added twice a week) and the colony number was counted. Data (n = 4, BMP9; n = 8, Dm) are displayed as percentage of control cells (mean ± S.E.M). **D.** Previously generated non-silencing (N.S.), shBMP9#1 and #2 stable HepG2 cell lines were plated in soft agar and counted after 3 weeks. Data from 4 experiments, displayed as percentage of N.S. cells (mean ± S.E.M). Statistical analysis was carried out using paired *t*-test and data were compared to untreated N.S. or control samples or as indicated, * = *P*<0.05, ** = *P*<0. 01, *** = *P*<0.001.

While studying the growth stimulatory effects of BMP9, we found that HepG2 cells were capable of proliferating in low serum (0.1% FBS) conditions (figure 1E and figure S2B in File S1). These findings, together with the fact that human transformed and non-transformed hepatocytes express BMP9 [20], [22], prompted us to speculate on a potential autocrine BMP9 effect in HepG2 cells. We first assayed BMP9 production in HepG2 cells by using a previously published sensitive bioassay for BMP9 [31], and we found that bioactive BMP9 is secreted by HepG2 cells into the culture media (data not shown). In a next step, we took advantage of the BMP inhibitors and ligand traps that have become available in recent years. Dorsomorphin (Dm) was discovered in a high throughput small molecule screen in zebrafish embryos as an inhibitor of the BMP type I receptors, i.e. ALK2, 3 and 6 [32]. LDN193189 is a BMP inhibitor that was developed using Dm as template and that has been described to be more potent and specific than Dm [33]. Both Dm (1 µM) and LDN193189 (100 nM) completely blocked phosphorylation of Smad1,5,8 triggered by BMP9 in HepG2 cells (figures 3A and 3B). In these conditions, Dm and LDN193189 effectively impaired BMP9-induced HepG2 cell growth (figures 3D and 3E). We then tested a purified Fc-coupled extracellular domain of ALK1 (ALK1ecd), which binds to BMP9 with high affinity [34], and has been used before as a specific ligand trap for BMP9 [27], [35], [36]. Incubation with ALK1ecd efficiently inhibited both BMP9-induced Smad1,5,8 phosphorylation and increase in HepG2 cell number, demonstrating that ALK1ecd acts as a potent inhibitor for BMP9 (figures 3C and 3F). Interestingly, we also observed that ALK1ecd treatment of Huh7, Hep3B and HepG2 cells growing in presence of 10% FBS resulted in reduced proliferation rates in these cell lines, suggesting that serum-derived BMP9 functions as a proliferative factor for them (figure S4 in File S1). On the other hand, THLE3 cell growth was not altered when ALK1ecd was added, further confirming that normal hepatocytes do not respond to BMP9 by increasing their proliferation (figure S4 in File S1). When HepG2 cells were incubated in low serum media and in the presence of either Dm or LDN193189 or importantly, ALK1ecd, we found a decrease in basal cell proliferation (figure 3G), data that may imply that autocrine BMPs, and specifically BMP9, contribute to basal HepG2 cell growth. Strikingly, when THLE3 were incubated in low serum media, cell number was not modified regardless the presence of ALK1ecd, suggesting that non-transformed hepatocytes do not respond to a putative autocrine BMP9 loop in terms of proliferation (figure 3H). To further confirm these data, we performed stable knockdown experiments: a non-silencing control (LMP-NS) and BMP9 shRNA pMir-based retroviral vectors (LMP-shBMP9#1 and #2) were used to generate stable HepG2 cell lines with reduced BMP9 levels (figure 4A). shBMP9 #1 and #2 HepG2 cell lines showed an impaired basal proliferation in 0.1% FBS media when compared to non-silencing control cells (LMP-NS) (figure 4B). Importantly, recombinant BMP9 was able to rescue this effect and triggered a strong growth response in cells expressing low levels of endogenous BMP9 (figure 4B). In agreement with these data, transient knockdown of BMP9 using siRNA yielded a 30% decrease in HepG2 cell number when cultured in low serum. Exogenously added BMP9 had a marked growth effect in these cells, highlighting once again a relevant role for BMP9 in HepG2 proliferation (figure S5 in File S1).

Since BMP signaling has been previously involved in anchorage independent growth in cancer cells including HCC cells [17], we next aimed to analyze the potential involvement of BMP9 in this process. In order to do so, colony formation assays were performed in HepG2 cells treated with BMP9. An increase in the number of colonies was observed upon BMP9 treatment when compared with the control condition (figure 4C). Conversely, when dorsomorphin (Dm) was assayed, we found a reduction in the number of colonies in Dm-treated cells as compared with non-treated cells (figure 4C). Furthermore, previously generated shBMP9 #1 and #2 stable HepG2 cell lines grown in anchorage independent conditions gave rise to a significantly reduced colony number compared with control (LMP-NS) cells (figure 4D). Taken together, these findings indicate that HepG2 cells exhibit an autocrine BMP9 signaling, which supports their growth, both in anchorage dependent and independent conditions.

### 4. BMP9 Increases Proliferation and Impairs Low Serum Triggered Apoptosis in HepG2 Cells

**Figure 5:**
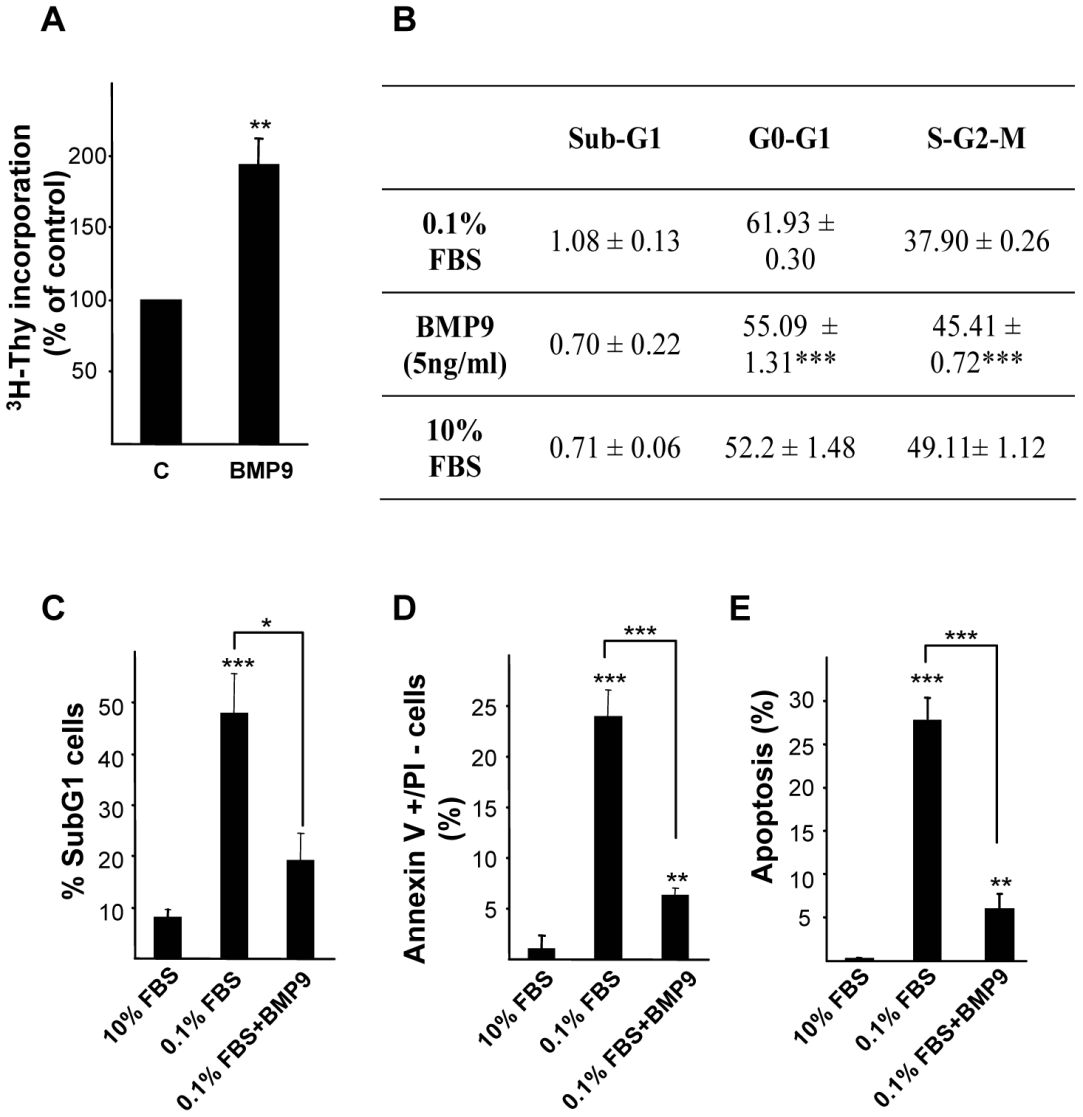
BMP9 increases proliferation and impairs low serum triggered apoptosis in HepG2 cells. **A.** DNA synthesis as determined by thymidine incorporation in HepG2 cells cultured for 24 hours in the absence or presence of BMP9 (5 ng/ml). Data are mean ± S.E.M. of 4 independent experiments and are displayed as percentage of untreated cells. **B.** HepG2 cells were incubated with or without BMP9 (5 ng/ml) in 0.1% FBS media or in the presence on 10% FBS media for 24 hours and then nuclear DNA content was analyzed by flow cytometry. Percentages of cells corresponding to the different cell cycle phases are shown. Data from 3 independent experiments performed in triplicate (mean ± S.E.M.). Statistical analysis was carried out using the paired *t*-test and data were compared to untreated samples (0.1% FBS), * = *P*<0.05, ** = *P*<0. 01, *** = *P*<0.001. **C, D, E.** HepG2 cells were treated as in **B** for 4 days. **C–D.** Cells were trypsinized and **C.** Nuclear DNA content was analyzed by flow cytometry. Percentages of hypodiploid (apoptotic) cells are shown. Data from 3 independent experiments performed in triplicate (mean ± S.E.M.). **D.** Cells were incubated with annexin V and PI. Subsequently, fluorescence intensity was measured in a FACScan flow cytometer and the percentage of annexin V positive/PI negative cells was calculated. Data from 3 independent experiments performed in triplicate (mean ± S.E.M.). **E.** Apoptotic nuclei were visualized and counted after PI staining under a fluorescence microscope. A minimum of 1000 nuclei was counted per condition. Data from 2 independent experiments performed in triplicate (mean ± S.E.M.). Statistical analysis was carried out using the paired *t*-test and data were compared to 10% FBS media treated samples or as indicated, * = *P*<0.05, ** = *P*<0. 01, *** = *P*<0.001.

So far our data clearly demonstrated that exogenously added, serum derived and autocrine derived BMP9 increases cell growth in HepG2 cells. Both proliferative and anti-apoptotic properties have been described for BMP signaling, and indeed BMP9 has been shown to increase proliferation in a variety of cell types [27], [34], [37], including liver cells [30]. In agreement with previous reports we found that BMP9 increased cell proliferation, measured by the [^3^H]-thymidine incorporation assay (figure 5A). We further confirmed these data by performing flow cytometric analysis of DNA content in HepG2 cells treated or not with BMP9 for 24 hours in 0.1% FBS media. Our data show that upon BMP9 treatment an increase in the percentage of HepG2 cells in S/G2/M phases is observed concomitantly with a decrease in the percentage of cells in G0/G1 phases. Indeed, cells incubated in presence of serum (10%) and BMP9 treated cells (0.1% FBS) present similar percentages of cells in each cell cycle phase, indicating that BMP9-promoted cell proliferation is comparable to that observed in cells incubated with 10% FBS (figure 5B). It is noteworthy that at 24 hours of treatment, the percentage of hypodiploid cells (sub-G1 peak) was very low and similar in all conditions assayed (percentage of subG1 cells ≤1%) indicating the absence of an apoptotic process in this early time point. It has been previously described that serum starvation triggers an apoptotic death in HepG2 cells [38], [39], effect that was also observed in our assay conditions (0.1% FBS). Indeed, after 4 days in low serum media an important increase in apoptotic cells was observed, measured by analysis of hypodiploid cells, by phosphatidylserine exposure on the outer leaflet of the plasma membrane and by quantification of apoptotic nuclei (figure 5C, 5D and 5E). Strikingly, BMP9 treatment efficiently abolished the apoptotic cell death in these conditions. In this line of evidence, we also found that BMP9 partially rescued cell death induced by other apoptotic stimuli such as TNF-α in HepG2 cells (figure S6 in File S1). Collectively, data presented here indicate that BMP9 has a dual role, both promoting a marked proliferative response and exerting an anti-apoptotic function in HepG2 cells, which results in a robust BMP9 effect on liver cancer cell growth.

### 5 BMP9 Expression is Increased over Normal Levels in 43% of HCC Human Samples

**Figure 6:**
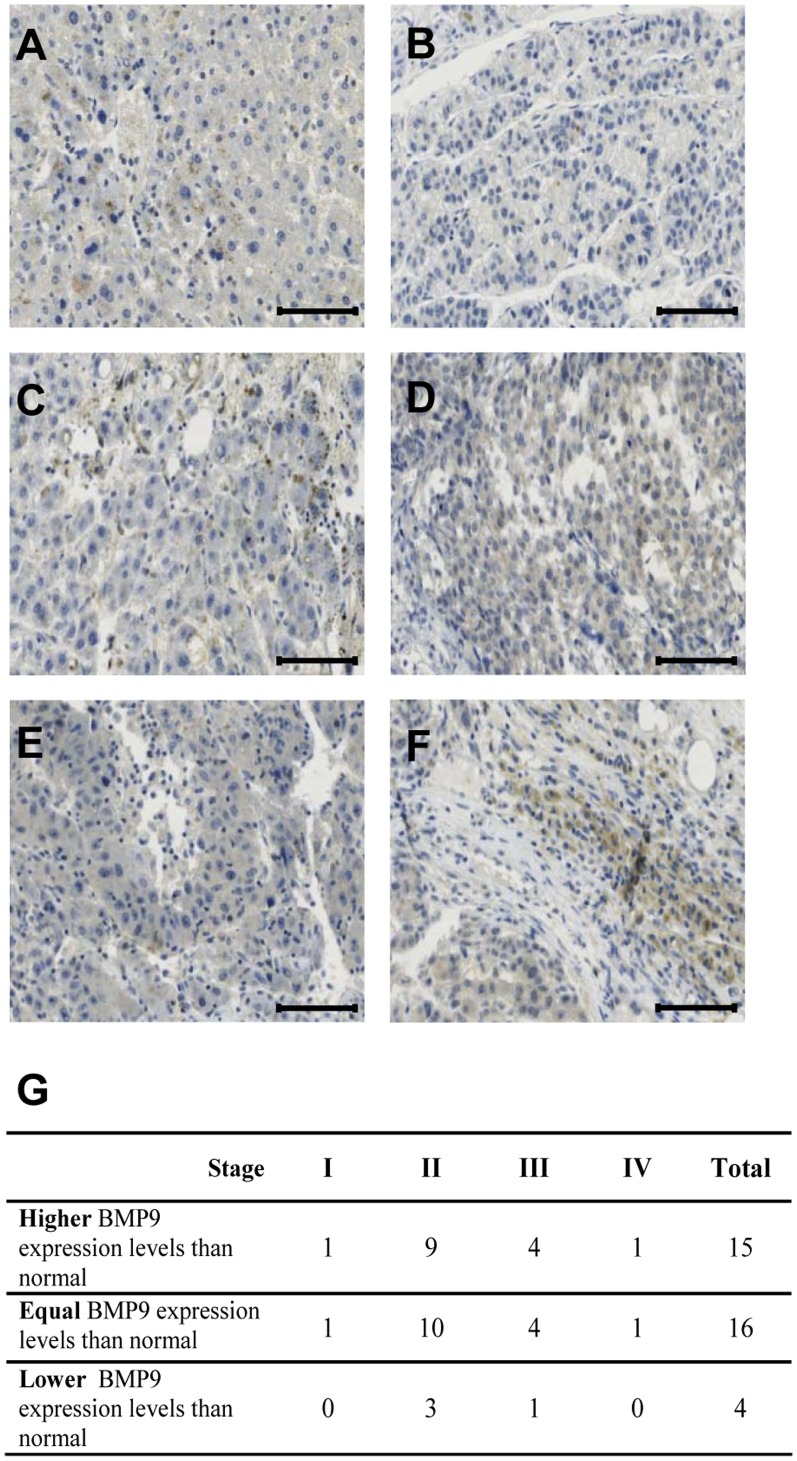
BMP9 expression is increased over normal levels in 43% of human HCC samples. Sections of formalin fixed paraffin embedded human liver tissues were stained with BMP9 antibody and counterstained with haematoxylin. Representative images of **A.** non neoplastic human liver; **B** and **C.** human HCC japanese stage II; **D, E, F.** HCC japanese stage III. Scale bars represent 100 µm. **G.** Table summarizing BMP9 IHC of a human liver cancer TMA. Staining was scored as stronger, equal and lower BMP9 staining than non-neoplastic tissue.

In order to explore BMP9 expression levels in human HCC samples, an immunohistochemistry (IHC) analysis on a small commercial liver cancer tissue microarray (TMA) was performed. The TMA contained 8 non-neoplastic and 35 HCC samples from cancer patients. As a negative control for BMP9 staining we used non-epithelial ovarian tumor samples, since we had previously shown that BMP9 expression is specifically absent in this ovarian cancer type [27] (figure S7 in File S1). Results presented in figure 6 show that BMP9 expression was detected in non-neoplastic liver samples (figure 6A and data not shown). These data are in agreement with previous data in the literature describing that normal human liver parenchymal cells i.e. hepatocytes and intrahepatic biliary epithelial cells express BMP9 [22]. When BMP9 staining was analyzed in HCC samples, we could observe different degrees in BMP9 expression, ranging from low expression (figures 6B and 6E) to high expression (figures 6C, 6D and 6F). We then classified HCC samples according to their BMP9 expression as compared to BMP9 expression levels in non-neoplastic liver specimens as follows: BMP9 expression levels over normal liver levels, BMP9 expression levels equal or lower than normal liver (figure 6G). Using this approach, we found that 43% (15 out of 35 HCC patients) presented increased BMP9 expression levels as compared with non-neoplastic liver. These results are in agreement with a recently published work that found that 39% of human HCC samples analyzed presented a moderate to strong BMP9 staining. Expression levels of BMP9 were positively correlated with invasion [23]. The number of samples contained in our TMA and their associated clinical data did not allow us to establish further clinical correlations. It would be most interesting to extend these observations with a more numerous TMA with matching clinical parameters. Nevertheless, these data suggest that BMP9 protein expression may be dysregulated during hepatocarcinogenesis and that elevated BMP9 production together with a differential response to this factor may constitute a strategy to increase the tumor cell proliferation and survival.

## Discussion

BMP9 regulates different cellular processes in a variety of cell types, and in particular, the BMP9/ALK1 axis has been unveiled as a major regulator of endothelial cell biology [35], [40], [41], [42]. An important role for BMP signaling in liver development is well established. However, only in the past few years the involvement of BMP signaling in adult liver homeostasis has been revealed [15]. In particular, the role of BMP9 in liver cells is only partially understood. In this regard, just recently has been demonstrated that BMP9 induces EMT that results in an increased migration of HCC cells [23]. Here, we have investigated the relevance of BMP9 in liver tumor cell biology and have found that BMP9 has dual pro-tumorigenic functions in HCC cells promoting both anchorage dependent and independent growth and survival.

The proliferative effect of BMP9 was only observed in transformed hepatocytes. Neither the immortalized human adult hepatocyte THLE3 cell line nor primary mouse adult or immortalized mouse neonatal hepatocytes did respond to BMP9 in terms of cell growth. Differences at receptor expression level could be the reason behind the different BMP9 response between non-transformed and transformed hepatic cells. Although we have not determined which type I receptor, ALK1 or ALK2, is mediating BMP9 responses several evidences point towards ALK2. Firstly, ALK1 expression is largely thought to be restricted to endothelial cells [43], although some ovarian cancer cells [27], chondrocytes [44] and interestingly, hepatic stellate cells [45] do express it. ALK1 mRNA was not detected by RT-PCR in HepG2 cells [23] (Herrera and Inman, unpublished data). Importantly, HepG2 cells do express ALK2 [23], [24], and data from our laboratory and others indicate that BMP9 is able to bind ALK2 receptor in non-endothelial cells, such as myoblasts, breast tumor cells and ovarian surface epithelial and ovarian cancer cells [27], [35]. It is well established that Dm and LDN193189 are type I BMP receptor (ALK2, ALK3 and ALK6) inhibitors, but no data are available in the literature regarding their potential inhibitory effect on ALK1. Data presented in this work indicate that both Dm and LDN193189 completely block BMP9-triggered signaling and proliferative effect in HepG2 cells, further suggesting ALK2 involvement in those events. Nevertheless, a detailed analysis of BMP receptors and also co-receptor expression in normal and transformed hepatocytes is required to fully clarify this point.

It is noteworthy that the differential response to BMP9 in immortalized human hepatocytes and HepG2 cells is independent of the BMP9 source. Our data indicate that exogenously added, serum-derived and autocrine BMP9 promotes cell growth in HepG2 cells but not in immortalized human hepatocytes. Both pharmacological inhibition of BMPs (dorsomorphin and LDN193189) and incubation of cells with ALK1ecd ligand trap in low serum conditions decreased HepG2 cell growth, while ALK1ecd treatment did not have any effect on THLE3 cell growth. Importantly, these results were confirmed by siRNA and shRNA-mediated BMP9 silencing, which further demonstrated that BMP9 knockdown in HepG2 cells decreased both basal proliferation and anchorage independent growth. Acquisition of growth factor autocrine loops have been described in HCC cells whose function is to enhance or support their tumorigenic properties. Thus, ligands of the EGF family and their receptors have been shown to play an active role not only in cell growth, but also in the regulation of cell motility and invasion [46], [47], [48], [49], [50]. HGF mRNA is not found in normal hepatocytes, but expressed in a high percentage of HCC, and in fact, *in vivo* data support a role for the autocrine HGF/Met axis in tumor promotion [51], [52], [53]. Along these lines, TGF-β inhibition by different means impairs HCC cell proliferation and invasion, suggesting a pro-tumorigenic role for autocrine TGF-β in HCC cells [54]. Different cancer types have been described to present BMP ligand autocrine loops, including BMP9 [12], [27]. Importantly, HCC cells overexpress BMP4 and BMP6, which are required for migration, invasion and anchorage independent growth [17], [18], [20]. In line with these evidences, our *in vitro* and *in vivo* data suggest that BMP9 production is increased in at least a subset of HCC and this autocrine loop enhances cell growth. How cancer cells acquire autocrine growth factors production is not completely understood. In the case of BMP9, our IHC data and previous reports indicate that healthy liver already produces BMP9 [22], [29], therefore, we hypothesize that HCC cells rather than acquire an autocrine production of BMP9 itself, gain the capacity of responding to BMP9 in terms of proliferation and cell survival.

Importantly, our data clearly show that BMP9 promotes cell growth at the same level of well-established liver growth factors such as EGF, IGF1 or insulin and is also involved in anchorage independent growth in HepG2 cells. BMP9 is not only a strong mitogen but it has also an important survival effect against low-serum-induced apoptosis. Thus, consistent with previous results [38], [39] serum deprivation triggers an apoptotic cell death in HepG2 cells that is significantly diminished when cells are treated with BMP9. Our data also reveal that BMP9 could have a survival effect in cell death induced by other apoptotic stimuli such as TNF-α. Taken together, these data constitute the first evidence for a role of BMP9 as an anti-apoptotic factor. The molecular mechanisms underlying such effect are the current focus of our studies.

In conclusion, awaiting further investigation to explore BMP9 function in non-transformed hepatocytes, we provide evidence to propose BMP9 as a regulator of HCC cell growth, by promoting proliferation and survival. Our data adds to the growing body of evidence that suggest the BMPs may have pro-tumorigenic roles in HCC and may be considered as potential therapeutic targets in HCC therapy. In this regard, several drug companies are developing ALK1 inhibitors on the basis of its antiangiogenic properties [43] and clinical trials to assess ALK1 inhibitors effects in advanced solid tumors have been launched. Giving the fact that HCC is a hypervascularized tumor [55], and that ALK1 is highly expressed in liver tumor blood vessels [56] HCC may be a good candidate for ALK1 inhibition therapeutic strategy. Furthermore, results presented in this work showing pro-tumorigenic functions for BMP9 in HCC cells acting to promote both anchorage dependent and independent growth and survival provide further evidences for the use of ALK1-fusion protein in HCC treatment considering that BMP9 withdrawal achieved by these drugs may target the liver cancer cell itself.

## Supporting Information

File S1 **Supplementary Information.**
(PDF)
