# Supplementary material for: BMP9 Is a Proliferative and Survival Factor for Human Hepatocellular Carcinoma Cells
Source: PLoS One. 2013 Jul 23;8(7):e69535. doi: 10.1371/journal.pone.0069535 (PMC3720667; doi:10.1371/journal.pone.0069535)
Supplement: File S1 — Supplementary Information. (PDF) [file pone.0069535.s001.pdf]

Supplementary information

Supplementary methods

Cell culture

SV40 immortalized mouse neonatal hepatocytes, a kind gift from Dr. Angela M. Valverde, were generated as previously described (Gonzalez-Rodriguez et al., 2007) and grown in DMEM supplemented with 10% FBS, 100 U of penicillin and streptomycin per ml.

TNF-α was from Peprotech (Neuilly-Sur-Seine, France) and cycloheximide from Sigma.

Hepatocytes isolation and culture.

Hepatocytes were isolated as previously described (Kao *et al.*, 1996). Briefly, livers from 3 month-old male mice were perfused with Hank's balanced salt solution supplemented with 10 mM Hepes and 0.2 mM EGTA for 5 min, followed by a longer perfusion (10-15 min) with William's medium E containing 10 mM Hepes and 0.03% collagenase type 1 (125 U/mg; Worthington). Livers were further minced, filtered through a 70µm cell strainer (BD) and viable hepatocytes were selected by centrifugation in Percoll, and seeded in collagen I-coated plates at a density of 25,000 cells/cm^2 in Dulbecco's modified Eagle Medium:F12 (1:1) supplemented with 10% FBS. Cells were kept overnight at 37ºC and 5% CO2, and used 12-16 hours later.

Cell viability assay

Celltitre-Glo™ luminescent cell viability assays (Promega, UK) were performed according to the manufacturer's instructions. Briefly, 1,000 cells/well in 96 well plates were plated and serum starved prior treatment with the different factors. Celltitre-Glo™ assays were then performed at the indicated time points by reducing media volumes to 50µl and adding 50µl of CellTitre-Glo™ reagent for 15 minutes. Readings were then taken on an OrionII microplateluminometer and analysed with Simplicity 4.2 software (Berthold, UK).

Transfection and siRNA knockdown

siRNA oligonucleotides were introduced into cells using HiPerFect transfection reagent (Qiagen) according to the manufacturer's protocol and cells were analyzed 48 hours after transfection. BMP9 siRNA oligonucleotides from Qiagen were used at 20nM concentration. As control (Non silencing) Allstars negative control (Qiagen) were used.

Supplementary figures legends

Supplementary figure S1

Primary mouse adult hepatocytes and immortalized mouse neonatal hepatocytes were incubated in the absence or in the presence of 1 or 5ng/ml BMP9 in 0% FBS (primary mouse hepatocytes) or 0.1% FBS (neonatal mouse hepatocytes) media and counted at day 2. Data from 2 independent experiments performed in triplicate (mean ± S.E.M.) are shown. Statistical analysis was carried out using the paired *t*-test and data were compared to untreated samples, * = *P*< 0.05, ** = *P* < 0. 01.

Supplementary figure S2

A. HepG2 cells were incubated with the indicated concentrations of BMP9 in 0.1% FBS media and CellTitre-Glo^{TM} cell viability assays were performed after 6 days of treatment (mean ± S.E.M, n=3). **B.** Proliferation curve of HepG2 cells incubated for the indicated time periods −/+ BMP9 (5ng/ml) in 0.1% FBS media and assayed by CellTitre-Glo^{TM} (mean ± S.E.M, n=3). Statistical analysis was carried out using the paired *t*-test and data were compared to untreated samples, * = *P*< 0.05, ** = *P* < 0. 01, *** = *P* < 0.001.

Supplementary figure S3

HepG2 cells were incubated with BMP9 (5ng/ml), insulin (INS, 100nM) IGF1 (10nM) and EGF (40ng/ml) in 0.1% FBS media or with 10% FBS media and counted after 4 days of treatment. Data from 3 independent experiments performed in triplicate (mean ± S.E.M.). Statistical analysis was carried out using paired *t*-test, * = *P*< 0.05, ** = *P* < 0. 01, *** = *P* < 0.001.

Supplementary figure S4

Huh7, Hep3B and HepG2 liver cancer cells and immortalized human hepatocytes (THLE3) were incubated −/+ ALK1ecd (16 F.M.E) in 10% FBS media and counted at day 4. Data from 2 independent experiments performed in triplicate (mean ± S.E.M.) are shown. Statistical analysis was carried out using paired *t*-test, * = $P< 0.05$, ** = $P < 0.01$, *** = $P < 0.001$.

Supplementary figure S5

HepG2 cells were transiently transfected with siRNA oligonucleotides. **A.** RNA was isolated after 48 hours. The levels of BMP9 were determined by quantitative RT-PCR and normalized to 18S. Data expressed relative to non silencing treated cells (assigned an arbitrary value of 1), from three determinations (mean ± S.D). **B.** Cells were treated as indicated for 6 days in 0.1% FBS media and counted. Data from 1 representative experiment performed in triplicate out of 2 independent experiments, displayed as percent of untreated non silencing transfected cells (mean ± S.D). Statistical analysis was carried out using paired *t*-test and data were compared as indicated, * = $P< 0.05$, ** = $P < 0.01$, *** = $P < 0.001$.

Supplementary figure S6

A. HepG2 cells were incubated for 15 hours in 0.1% FBS media in the absence or in the presence of TNF-α (15ng/ml) and cycloheximide (0.5μg/ml) and in the absence or presence 5ng/ml BMP9 and counted. Data from 5 independent experiments performed in triplicate (mean ± S.E.M.) are shown. Statistical analysis was carried out using paired *t*-test and data were compared to untreated samples or as indicated, * = $P< 0.05$, ** = $P < 0.01$, *** = $P < 0.001$.

Supplementary figure S7

Sections of formalin fixed paraffin embedded human ovary cancer tissues were stained with BMP9 antibody and counterstained with haematoxylin. Representative images of

non epithelial ovarian cancer samples: **A**) yolk sac tumor cells and **B**) dysgerminoma cells. Scale bars represent 100 μm.

	FORWARD	REVERSE
**Non silencing**	TCGAGAAGGTATATTGCTGTTGACAGTGAGCGATCTCGCTTGGGCGAGAGTAAGTAGTGAAGCCACAGATGTACTTACTCTCGCCCAAGCGAGAGTGCCTACTGCCTCGG	AATTCCGAGGCAGTAGGCACTTACTCTCGCCCAAGCGAGATTACATCTGTGGCTTCACTAACTCTCGCTTGGGCGAGAGTAAGCGCTCACTGTCAACAGCAATATACCTTC
**shBMP9#1**	TCGAGAAGGTATATTGCTGTTGACAGTGAGCGAAAGGACTGATTCAATCTGCATTAGTGAAGCCACAGATGTAATGCAGATTGAATCAGTCCTTGTGCCTACTGCCTCGG	AATTCCGAGGCAGTAGGCACAAGGACTGATTCAATCTGCATTACATCTGTGGCTTCACTAATGCAGATTGAATCAGTCCTTTCGCTCACTGTCAACAGCAATATACCTTC
**shBMP9#2**	TCGAGAAGGTATATTGCTGTTGACAGTGAGCGCCAACAGGTACACGTCCGATAATAGTGAAGCCACAGATGTATTATCGGACGTGTACCTGTTGTTGCCTACTGCCTCGG	AATTCCGAGGCAGTAGGCAACAACAGGTACACGTCCGATAATACATCTGTGGCTTCACTATTATCGGACGTGTACCTGTTGGCGCTCACTGTCAACAGCAATATACCTTC

Supplementary Table S1

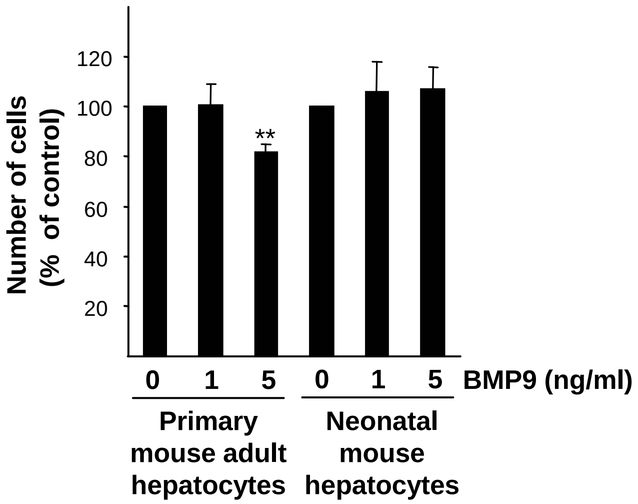

Supplementary Figure S1

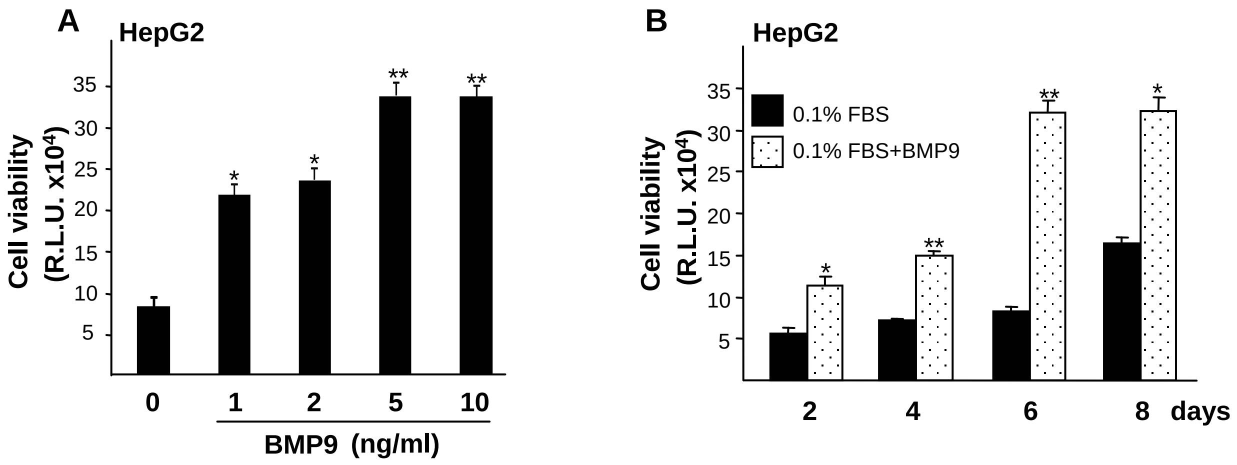

Supplementary Figure S2

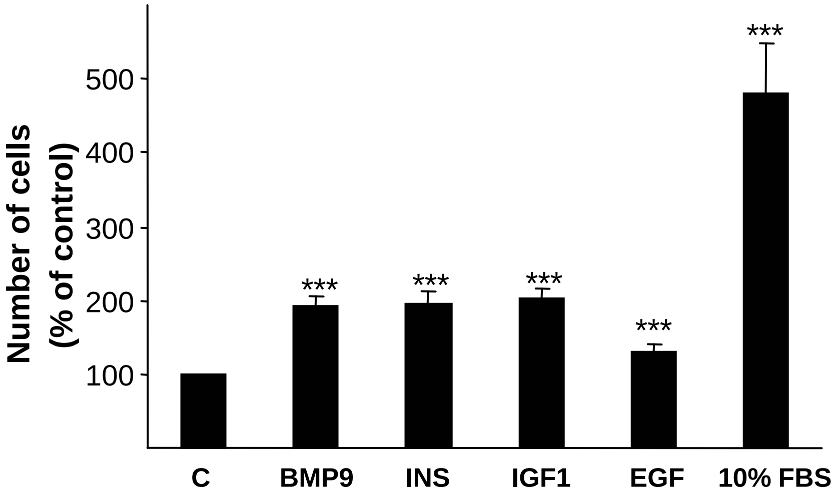

Supplementary Figure S3

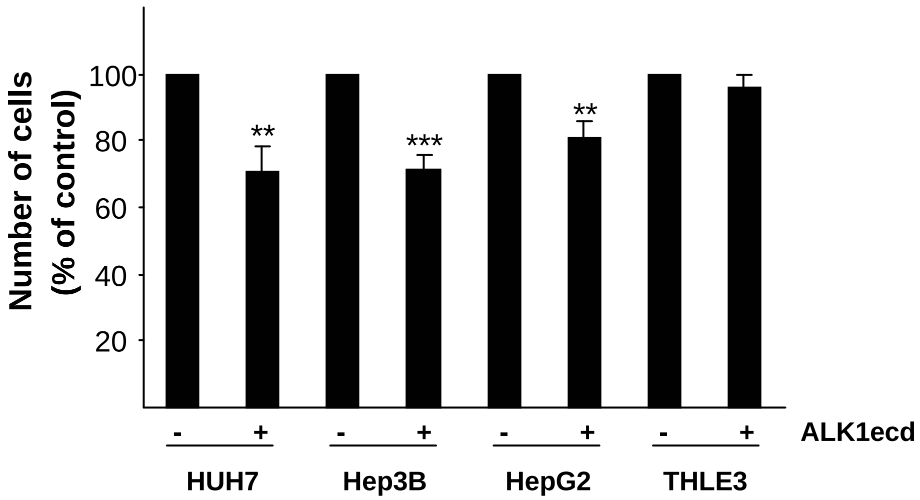

Supplementary Figure S4

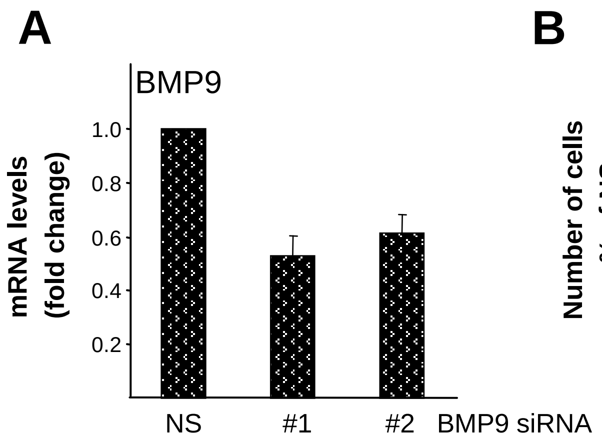

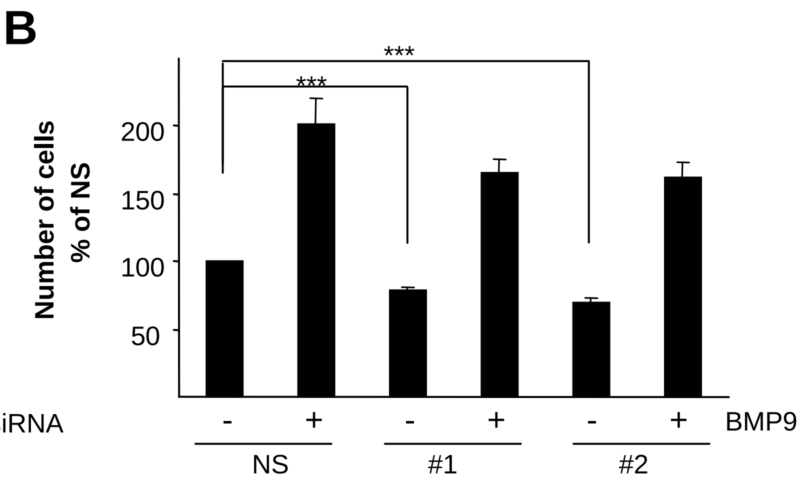

Supplementary Figure S5

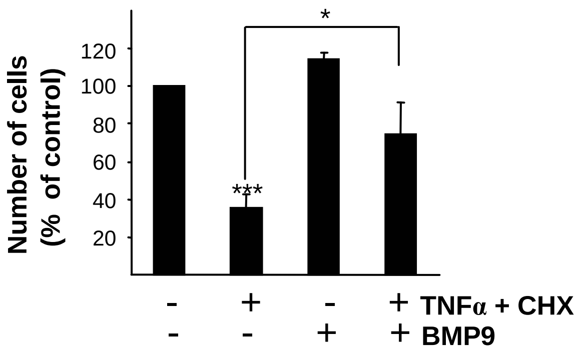

Supplementary Figure S6

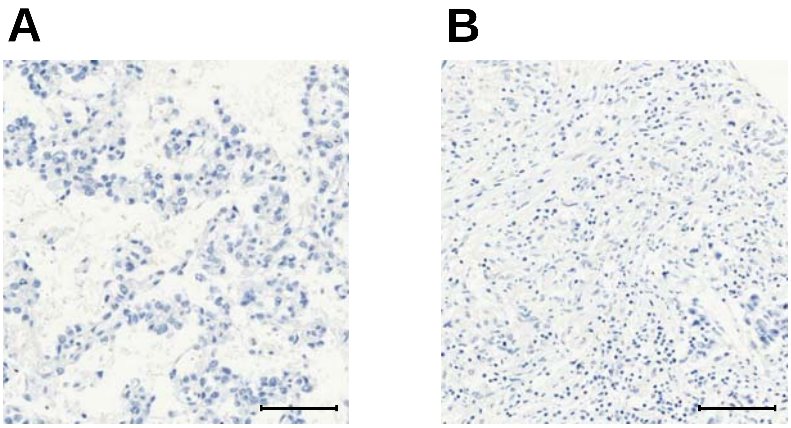

Supplementary Figure S7
